# Fracture Resistance of CAD/CAM Lithium Disilicate of Endodontically Treated Mandibular Damaged Molars Based on Different Preparation Designs

**DOI:** 10.1155/2019/2475297

**Published:** 2019-05-12

**Authors:** Carolina Clausson, Cristiano Clausson Schroeder, Paulo Vicenti Goloni, Flavio Artur Rego Farias, Leandro Passos, Raquel Virgínia Zanetti

**Affiliations:** ^1^Department of Prosthetic Dentistry, Dental School and Institute and Research Center São Leopoldo Mandic, Rua Doutor José Rocha Junqueira 13, Campinas, São Paulo 13045-755, Brazil; ^2^Department of Implant Dentistry, Dental School and Institute and Research Center São Leopoldo Mandic, Rua Doutor José Rocha Junqueira 13, Campinas, São Paulo 13045-755, Brazil; ^3^Department of Prosthetic Dentistry, Federal Fluminense University, Health Institute of Nova Friburgo, School of Dentistry, Rua Doutor Silvio Henrique Braune 22, Nova Friburgo, Rio de Janeiro 28625-650, Brazil; ^4^Department of Dentistry, University of Alberta, Edmonton Clinic Health Academy, Faculty of Medicine and Dentistry, School of Dentistry, 11405-87 Ave NW, Edmonton, Canada T6G 1C9

## Abstract

The aim of this study was to evaluate the fracture resistance of 2 different types of all-ceramic crown using immediate dentin sealing (IDS), obtained using a CAD/CAM system on molars with different preparations. Forty extracted lower molars were endodontically treated and divided into four groups (n = 10) according to the dental preparation. Group 1 (SP0) was prepared without filling the pulp chamber and crown-root junction was located at the cementoenamel junction (CEJ). Group 2 (SP1) was prepared without filling the pulp chamber and crown-root junction was located 1-mm above the CEJ. Groups 3 and 4 contained a flat preparation surface with no axial wall height. Group 3 (CP0) was made IDS with complete filling of the pulp chamber with composite resin and crown-root junction was located at the CEJ. Group 4 (CP1) was prepared with complete filling of the pulp chamber and crown-root junction was located 1-mm above the CEJ. All groups were restored with CAD/CAM lithium disilicate ceramic crowns. Specimens were subjected to the fracture test and statistically analyzed using analysis of variance (ANOVA). Fracture mode was determined using a stereoscopic microscope, classified as repairable or nonrepairable, and analyzed using Fischer's exact test. Results indicated that there were no significant differences between the groups in terms of fracture resistance or fracture pattern (p >0.05). Fracture resistance was the lowest in the SP0 group, followed by the SP1 group (1634.38 N) of CP0 (1821.50 N), and it was the highest in the CP1 group. There was a predominance of nonrepairable fractures and there were no significant differences in the fracture resistance and fracture mode of CAD/CAM lithium disilicate molar all-ceramic crowns. Endodontically treated molars teeth might be restored with endocrowns or all-ceramic crowns on flat preparation; however tooth fracture failures that affect reliability of these types of restorations should be considered.

## 1. Introduction

Endodontically treated teeth with reduced structure present a higher risk of mechanical failure than vital teeth [1–5]. Currently, an alternative approach for reconstructing teeth with significant loss of structure and endodontically treated is the usage of endocrown, a dental crown that has an anchorage and additional adhesion in the pulp chamber, which eliminates the need to use root posts [6, 7]. The advantages of endocrown restorations include minimally invasive approach, lower cost, and clinical time than conventional core and crown restorations [7–11]. 

The most common dental preparation technique for endocrowns is the use of the pulp chamber as an additional retention form. For this, a preparation is needed, to cause expansion of the walls, resulting in even greater loss of tooth structure. Another alternative, to avoid this loss, is to fill the pulp chamber with composite resin [12, 13]. 

Nowadays, three types of endocrowns were described: Class 1 describes a tooth preparation where at least two cuspal walls have a height superior to the half of their original height. Class 2 describes a tooth preparation where maximum one cuspal wall has a height superior to the half of its original height. Class 3 describes a tooth preparation where all cuspal walls are reduced for more than the half of their original height [11].

Another preparation has been described as flat surface preparation with no axial wall height, with no pulp chamber anchorage, since adhesive strategies have become more and more reliable, and dental preservation has been searched [14–16].

However, there is limited information regarding the evaluation of mechanical properties of these all flat preparations with complete filling of the pulp chamber with composite resin (IDS) compared to the technique that uses the pulp chamber for additional retention. The aim of this study was to evaluate the fracture resistance and fracture mode of all-ceramic crowns prepared either with or without complete filling of the pulp chamber with composite resin, and the crown-root junction located either at the CEJ or 1-mm above the CEJ.

The null hypotheses were the following: (1) there would be no difference in maximum fracture load between the different preparations and (2) the different preparations would not induce fractures below the CEJ, which were classified as nonrepairable.

## 2. Materials and Methods

### 2.1. Sample Selection

The current experimental study was performed using human mandibular molars without root caries, fillings, restorations, previous endodontic treatments, or cracks at 2x magnification, which may affect their fracture resistance to loading. Forty teeth were selected based on visual examinations of complete root formation and presence of a crown with four cusps and cruciform sulci. These anatomical characteristics are similar to those of second lower molars and teeth with simple and easily reproducible anatomy, given that this anatomical restoration pattern favors the uniform distribution of axial loads. A single operator performed all the procedures. The Ethics Committee of the São Leopoldo Mandic Institute and Center for Dental Research approved the current study (Protocol number 1.049.832). The specimens were disinfected in 0.2% Timol solution for 48h and stored in normal saline.

### 2.2. Tooth Preparation and Root Canal Filling

The teeth preparation started by using an electric motor (EM-E6 TP, W&H) and a hand piece (Synea WA-99LT, W&H) with a diamond bur (3069, KG Sorensen) at 3,000× speed under constant water irrigation at 2.5× magnification to remove horizontally the coronary portion of the tooth at the CEJ in 20 specimens and 1-mm above the CEJ in the other 20 specimens. An access cavity was prepared using a diamond bur (1016, KG Sorensen) and teeth were prepared using a sequence of files (K-Flex, Kerr Corporation) according to the manufacturer's instruction. Irrigation was performed using 5.25% sodium hypochlorite solution. Teeth were filled with gutta-percha cones (Dentsply Maillefer) and zinc oxide eugenol-based endodontic filling cement (Endofill, Dentsply Maillefer). The filling cement of all the specimens was removed in the coronary third and a eugenol-free temporary filling material was used for temporary restoration for 7 days before performing the adhesive procedures. The crown-root junction was located at the CEJ in 20 specimens and 1-mm above the CEJ in 20 specimens. To standardize the restoration's extension in the pulp chamber, for each group of 20 specimens, ten teeth with a chamber size of at least 2 mm were selected, and the chambers of the remaining ten specimens in each group were completely filled with composite resin. Thus, four groups (n = 10) were obtained, as shown in Table 1 [8, 9, 17].

### 2.3. All-Ceramic Crowns Preparation

#### 2.3.1. Groups SP0 and SP1

Pulp chambers were completely filled with composite resin (Filtek Z350 XT, 3M ESPE) using the incremental technique to seal the canal and standardize the depth of the preparation used just in thin layer thickness. The treatment involved applying a self-etch adhesive (Clearfil SE Bond, Kuraray) for 20 seconds and then a mild oil-free air jet and curing with a high-power LED curing light (Bluephase N, Ivoclar Vivadent) for 15 seconds at 1200mW/cm^2^. The internal angles were rounded off and the post walls, where present, were removed by a single operator using high-speed diamond tips (4137, KG Sorensen).

#### 2.3.2. Groups CP0 and CP1

**Figure 1:**
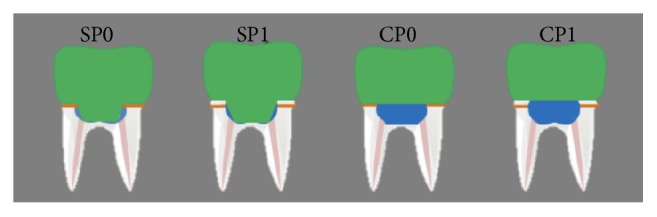
Schematic representation of the restorative strategies. Green, CAD/CAM restoration; blue, composite resin; pink, endodontic filling; and orange: CEJ.

The specimens received the same adhesive treatment as SP0 and SP1, and the pulp chambers were completely filled with composite resin (Bulk Fill Surefil SDR Flow, Dentsply Sirona). After filling, the specimens in both groups were polished using fine-grained and extra-fine-grained diamond tips and abrasive rubbers (composite polisher Politip F, Ivoclar Vivadent) (Figure 1).

### 2.4. Restorations' Design

The crown restoration for tooth 37 was selected using the Biogeneric Copy design mode of the CAD/CAM software (Cerec 4.4.4, Sirona Dental Systems). Video images were acquired using a CAD/CAM system (Omnicam, Sirona Dental Systems, Bensheim, Germany) for the Biogeneric Copy crown restoration (healthy second mandibular molar) and dental preparation.

The two models were correlated allowing the restoration design to have the same dimensions as the integrated tooth 37 previously scanned. All dental preparations were correlated with a unique Biogeneric Copy to standardize the occlusal anatomy and coronal height design of all restorations. The steps performed within the software for virtual building of the crown are detailed below.

The model axis was determined by positioning the models according to the mid-line, inclination, and alignment of the anterior teeth. The margin was homogeneously delineated. The Insertion Axis was defined and presented no undercut areas and the Copy Line was determined allowing a standard crown design. Restoration Parameters were set as follows: Radial Spacer, 80*μ*; Occlusal Spacer, 80*μ*; Occlusal Milling Offset, 0*μ*; Proximal Contacts Strength, 0*μ*; Occlusal Contacts Strength, 25*μ*; Dynamic Contact Strength, 25*μ*; Minimal Thickness (Radial), 800*μ*; Minimal Thickness (Occlusal), 800 *μ*; Margin Thickness, 50 *μ*. No design modifications were done in any sample and the sprue was positioned at the lingual surface of the crown.

### 2.5. All-Ceramic Crowns Fabrication

Forty monolithic crowns were fabricated by milling ceramic blocks of lithium disilicate glass-ceramic (e.max CAD blocks HT, shade A3 on Vitapan, 14-mm long, LOT: U03248, Ivoclar Vivadent). Restorations were milled with a four-axial milling unit (Cerec MCXL, Sirona Dental Systems, Bensheim, Germany) in a one-step mode, using a Step Bur 12S (Sirona Dental Systems, Bensheim, Germany) and a Cylindrical Pointed Bur 12S (Sirona Dental Systems, Bensheim, Germany). Cutting diamonds were changed after milling twelve crowns. After the milling process, a diamond bur was used to remove the restoration's sprue with water spray used as a coolant.

The adaptation of all-ceramic crowns was checked visually at the margins and internally with liquid silicone (Oranwash, Zhermack SpA). Specimens were discarded in case of misfit. Subsequently, samples were crystallized in a ceramic furnace (Atlantis Pro, Kota) according to the manufacturer's instructions and firing protocols. A single operator performed all the procedures.

### 2.6. Cementation

The internal surfaces of lithium disilicate glass-ceramic restorations were treated with 9.5% hydrofluoric acid for 20 seconds. The etched internal surfaces of all crowns were cleaned using a water spray, followed by ultrasonic cleaning (Easyclean, Renfert GmbH, Germany) in distilled water for 60 seconds. All restorations were dried for 20 seconds, and a silane (Monobond-S, Ivoclar Vivadent) was applied to the internal surfaces of the crowns (as per manufacturer's recommendations). Then, the restorations were air dried for 5 seconds. Teeth surfaces were treated with 37% phosphoric acid (N-Etch, Ivoclar Vivadent) for 15 seconds, cleaned using a water spray with for 60 seconds, and gently dried for 10 seconds. An adhesive (Scotchbond Universal Adhesive, 3M ESPE) was applied to the enamel and dentin (as per manufacturer's recommendations).

A resin cement (Variolink II, Ivoclar Vivadent) was used in the dual-curing mode and applied to the internal surface of teeth and restorations; the crowns were then seated on each tooth preparation and held in position by exerting constant pressure of 6N (750g) for 5 minutes [18].

Gross excess material was removed using an explorer (EXD 5, Hu-Friedy), and the cementation interface was covered with an oxygen protective gel (Air Block Liquid Strip, Ivoclar Vivadent), followed by 20 seconds of light polymerization in each face of the crown using a light-curing device in Hi-Power mode (Bluephase N, Ivoclar Vivadent) at 1200 Mw/cm^2^.

### 2.7. Fracture Test

Samples were introduced in cylindrical PVC rings and embedded 2 mm beneath the CEJ using auto polymerized colourless acrylic resin (Classico Jet, Dencor). All specimens were stored at 100% humidity and 37°C for 24 hours prior to the fracture test.

**Figure 2:**
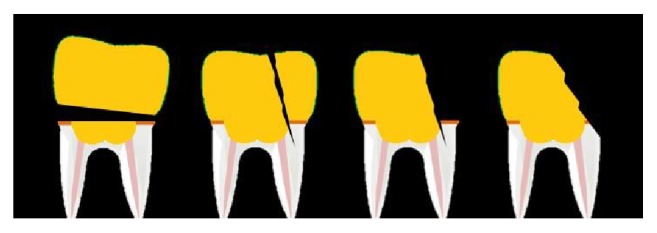
Schematic representation of the fracture patterns. (1) Detachment of the crown without fracture; (2) fracture without detachment of the crown and presence of a crack in the remaining tooth structure; (3) fracture with partial detachment of the crown and presence of a crack in the remaining tooth structure; and (4) fracture with partial detachment of the crown and the remaining tooth structure.

A clamp was placed at the base of a universal testing machine (EMIC DL 2000, INSTRON) and the load transferred in a test probe (6mm diameter) that rested on the central fossa of the all-ceramic crowns with a crosshead speed of 1 mm/min until the fracture and/or tooth and/or crown detachment occurred. Fracture loads were recorded in Newtons (N) and specimens were examined using an optical light microscope (EK3ST, Eikonal Optical and Analytical Equipment) at 40x magnification to determine the predominant failure pattern. Failures were classified as repairable (type 1) or nonrepairable (types 2, 3, or 4) (Figure 2).

Fracture test data were analyzed with statistical software (SPSS version 23.0, SPSS Inc.) using a one-way analysis of variance (ANOVA) (! = 0.05). Fracture pattern was classified as repairable or nonrepairable according to the fracture characteristics and data were analyzed using Fischer's exact test.

## 3. Results

Fracture load data and statistics are presented in Table 2. The mean fracture resistance varied from 1546.29N to 1924.05N. Resistance was lower in SP0 (1546.29N) and higher in CP1 (1924.05N). However, regarding the fixed margin of error (5%), there were no significant differences between the groups (p > 0.05).

The comparison of the fracture mode between the groups indicated that the highest difference occurred in the CP0 group, with five cases in the CP0 group and no cases in the SP1 group. Nonetheless, these differences were not significant (p > 0.05) (Table 3).

One specimen presented an adhesive failure (repairable) while others presented nonrepairable failure on the restoration and remaining tooth structure.

## 4. Discussion

Under such these* in vitro *circumstances, endocrowns are benefited by the advances in adhesive materials, resin cement, and acid-sensitive ceramic materials, as used in other studies [9, 19]. For many years, teeth with significant loss of structure have been treated with intraradicular posts, which promote higher tooth wear, reducing tooth resistance [20, 21].

The current endocrown concept is based on a cavity design that preserves the maximum amount of tooth surface for cementation as long as retentive areas are no longer a prerequisite [9, 10, 22]. The choice to use lower second molars in this study was based on the occlusal anatomy of these teeth; the uniform axial load distribution presented in other studies [23, 24] and preparations were based on conventional endocrown's design and all flat preparation [6–10, 12–17, 22, 24–26]. The transfer of stress between the restoration and tooth is mediated by the resin cement.

The occlusal anatomy, thickness, shape, and slope of the restorations' cusps were standardized by the Biogeneric Copy design of the CAD/CAM software, which standardized the loading point application through mechanical test. However, one limitation of this study is that the load was applied only axially [9, 24]. Previous studies tested biomechanical characteristics of endocrowns and presented survival rates improved by using an oblique compressive load. Ceramic endocrowns protected the remaining tooth structure because of their high modulus of elasticity; nonetheless, this feature favored cementation failure [27]. Regarding the stress distribution, other studies accomplish the indication of a more flexible restorative material, such as composite, in smaller dimensions [28]. It is known that the highest maximum bite force is exhibited in the molar region [29]. Unilateral measurement of maximum bite force in the molar region averages between 300 and 600 Newtons (N) in healthy adults with natural teeth [30, 31]. If the force is measured bilaterally in the molar region, the recorded force is about 40% higher than the unilateral measurement [32, 33].

Although it is difficult to accurately determine occlusal forces because of the high number of variables, some authors [24] reported no significant differences between conventional ceramic endocrowns and crowns over composite resin fillings, and, considering the masticatory load values reported in previous studies [18, 34–36] and the average fracture load of groups in this study, it may be surmised that such restorations may not be capable of complications/failures related to fracture strength.

Other studies also indicate endocrowns as a potentially restoring application in endodontically treated teeth, presenting better results than conventional core and post-crown restorations [8, 9, 11, 25, 37], despite the predominance of catastrophic failure when subjected to load tests [8, 22, 26, 35]. Some authors [17] also found no significant differences between endocrowns and conventional crowns. Although studies using flat preparations [14, 15] have shown basically adhesive failures that presented lower values, in this study only 1 specimen showed adhesive failure (CP0 group), which was repairable and explainable because of enamel's absence. In all other tests samples' failures were irreparable presenting similar result to another study [26] and contrasting to other literature results.

According to the previous discussion and results, the first null hypothesis was accepted, since there were differences in maximum fracture load between the different preparations but not statistically significant. The second null hypothesis was also accepted, since the comparison of the fracture mode between the groups indicated differences between the groups, but not statistically significant.

Finally, the intention was not to create over-resistant restorations with this study, but to search for a restorative technique that recovered compromised tooth structures with minimal wear of the remaining tooth structure as there is no need to enlarge root canals as is necessary for intraradicular posts when using endocrowns. Further* in vitro *and* in vivo *investigations should be performed, as the results of this study do not necessarily reflect the clinical performance of this type of restoration.

## 5. Conclusions

Within the limitations of this in vitro experiment, no statistically significant differences were found in the fracture resistance and fracture mode of CAD/CAM lithium disilicate molar endocrowns comparing to flat preparations. Regarding the fracture mode, nonrepairable fractures were relatively more common. Endodontically treated molars teeth might be restored with endocrowns; however tooth fracture failures that affect reliability of this type of restoration should be considered, and further studies involving fatigue studies as thermocycling and cyclic loading are recommended.

## Figures and Tables

**Table 1:** Distribution of groups according to type of restoration.

Group	Description

SP0	10 endocrowns without complete filling of the pulp chamber with composite resin and crown-root junction located at the CEJ.
SP1	10 endocrowns without complete filling of the pulp chamber with composite resin and crown-root junction located 1-mm above the CEJ.
CP0	10 all-ceramic crowns on flat preparation with complete filling of the pulp chamber with composite resin and crown-root junction located at the CEJ.
CP1	10 all-ceramic crowns on flat preparation with complete filling of the pulp chamber with composite resin and crown-root junction located 1-mm above the CEJ.

**Table 2:** Fracture test result (values are expressed in Newtons).

	Groups				
Statistics	SP0	SP1	CP0	CP1	*P* value

Mean	1546.29	1634.38	1821.50	1924.05	p (1) = 0.356
Minimum	785.83	882.38	1014.18	1164.30	
Maximum	2402.59	2462.50	2850.13	2654.20	

**Table 3:** Fracture mode by group (Fisher's exact test).

	Group									
Type of fracture	SP0		SP1		CP0		CP1		Group	*P* value
	n	%	n	%	n	%	n	%	Total	

1. Detachment of the endocrown without fracture	-	-	-	-	1	10.0	-	-	1	p (1) =0.170
2. Fracture without detachment of the endocrown and presence of a crack in the remaining tooth structure.	4	40.0	5	50.0	2	20.0	3	30.0	14	
3. Fracture with partial detachment of the endocrown and presence of a crack in the remaining tooth structure.	1	10.0	-	-	5	50.0	2	20.0	8	
4. Fracture with partial detachment of the endocrown and remaining tooth structure.	5	50.0	5	50.0	2	20.0	5	50.0	17	
Total	10	100.0	10	100.0	10	100.0	10	100.0	40	

## Data Availability

The data used to support the findings of this study are available from the corresponding author upon request.

## Supplementary Materials

Supplementary Materials Fracture types: (1) detachment of the endocrown without fracture; (2) fracture without detachment of the endocrown and presence of a crack in the remaining tooth structure; (3) fracture with partial detachment of the endocrown and presence of a crack in the remaining tooth structure; (4) fracture with partial detachment of the endocrown and remaining tooth structure.

## References

[1] Ferrari M., Vichi A., Mannocci F., Mason P. N. (2000). Retrospective study of the clinical performance of fiber post. *American Journal of Dentistry*.

[2] Morgano S. M., Rodrigues A. H. C., Sabrosa C. E. (2004). Restoration of endodontically treated teeth. *Dental Clinics of North America*.

[3] Sorrentino R., Salameh Z., Zarone F., Tay F. R., Ferrari M. (2007). Effect of post-retained composite restoration of MOD preparations on the fracture resistance of endodontically treated teeth. *The Journal of Adhesive Dentistry*.

[4] Dietschi D., Duc O., Krejci I., Sadan A. (2008). Biomechanical considerations for the restoration of endodontically treated teeth: a systematic review of the literature. Part II (Evaluation of fatigue behavior, interfaces, and in vivo studies). *Quintessence International*.

[5] Faria A. C. L., Rodrigues R. C. S., de Almeida Antunes R. P., de Mattos M. D. G. C., Ribeiro R. F. (2011). Endodontically treated teeth: characteristics and considerations to restore them. *Journal of Prosthodontic Research*.

[6] Bindl A., Mörmann W. H. (1999). Clinical evaluation of adhesively placed Cerec endo-crowns after 2 years—preliminary results. *The Journal of Adhesive Dentistry*.

[7] Manta G. F., Goyata F. R. (2010). Endocrown: uma alternativa restauradora para dentes posteriores desvitalizados: relato de caso clínico. *Revista de Dental Press Estética*.

[8] Chang C.-Y., Kuo J.-S., Lin Y.-S., Chang Y.-H. (2009). Fracture resistance and failure modes of CEREC endo-crowns and conventional post and core-supported CEREC crowns. *Journal of Dental Sciences*.

[9] Biacchi G. R., Basting R. T. (2012). Comparison of fracture strength of endocrowns and glass fiber post-retained conventional crowns. *Operative Dentistry*.

[10] da Cunha L. F., Gonzaga C. C., Pissaia J. F., Correr G. M. (2017). Lithium silicate endocrown fabricated with a CAD-CAM system: a functional and esthetic protocol. *Journal of Prosthetic Dentistry*.

[11] Belleflamme M. M., Geerts S. O., Louwette M. M., Grenade C. F., Vanheusden A. J., Mainjot A. K. (2017). No post-no core approach to restore severely damaged posterior teeth: An up to 10-year retrospective study of documented endocrown cases. *Journal of Dentistry*.

[12] Lander E., Dietschi D. (2008). Endocrowns: a clinical report. *Quintessence International*.

[13] Schlichting L. H., Machry L., Hilgert L. A.

[14] Spriggel R., DuVall N., Brewster J., Roberts H. (2018). Axial wall height effect on failure of adhesively luted computer assisted design/computer assisted manufactured ceramic crowns on preparations containing advanced total occlusal convergence. *Journal of Adhesion Science and Technology*.

[15] Miller M., DuVall N., Brewster J., Wajdowicz M. N., Harris A., Roberts H. W. (2018). Bicuspid axial wall height effect on CAD/CAM crown fracture mode on preparations containing advanced total occlusal convergence. *Journal of Prosthodontics*.

[16] Leprince J. G., Leloup G., Hardy C. M. F. (2016). Considerationsfor the restoration of endodontically treated molars. *The Guidebook to Molar Endodontics*.

[17] Guo J., Wang Z., Li X., Sun C., Gao E., Li H. (2016). A comparison of the fracture resistances of endodontically treated mandibular premolars restored with endocrowns and glass fiber postcore retained conventional crowns. *The Journal of Advanced Prosthodontics*.

[18] Egbert J. S., Johnson A. C., Tantbirojn D., Versluis A. (2015). Fracture strength of ultrathin occlusal veneer restorations made from CAD/CAM composite or hybrid ceramic materials. *Oral Science International*.

[19] Gré C. P., de Ré Silveira R. C., Shibata S., Lago C. T., Vieira L. C. (2015). Silanization effect on microtensile bond strength of a self-adhesive luting material to a disilicate-based glass ceramic. *RSBO*.

[20] Sorensen J. A., Martinoff J. T. (1984). Intracoronal reinforcement and coronal coverage: A study of endodontically treated teeth. *The Journal of Prosthetic Dentistry*.

[21] Dietschi D., Duc O., Krejci I., Sadan A. (2007). Biomechanical considerations for the restoration of endodontically treated teeth: a systematic review of the literature-Part 1. Composition and micro- and macrostructure alterations. *Quintessence International*.

[22] Magne P., Carvalho A. O., Bruzi G., Anderson R. E., Maia H. P., Giannini M. (2014). Influence of no-ferrule and no-post buildup design on the fatigue resistance of endodontically treated molars restored with resin nanoceramic CAD/CAM crowns. *Operative Dentistry*.

[23] Bindl A., Richter B., Mörmann W. H. (2005). Survival of ceramic computer-aided design/manufacturing crowns bonded to preparations with reduced macroretention geometry. *International Journal of Prosthodontics*.

[24] Hasan I., Frentzen M., Utz K.-H., Hoyer D., Langenbach A., Bourauel C. (2012). Finite element analysis of adhesive endo-crowns of molars at different height levels of buccally applied load. *Journal of Dental Biomechanics*.

[25] Sedrez-Porto J. A., Rosa W. L. D. O. D., da Silva A. F., Münchow E. A., Pereira-Cenci T. (2016). Endocrown restorations: a systematic review and meta-analysis. *Journal of Dentistry*.

[26] Hayes A., Duvall N., Wajdowicz M., Roberts H. (2017). Effect of endocrown pulp chamber extension depth on molar fracture resistance. *Operative Dentistry*.

[27] Zhu J., Rong Q., Wang X., Gao X. (2017). Influence of remaining tooth structure and restorative material type on stress distribution in endodontically treated maxillary premolars: a finite element analysis. *Journal of Prosthetic Dentistry*.

[28] Pedrollo Lise D., Van Ende A., De Munck J., Umeda Suzuki T. Y., Cardoso Vieira L. C., Van Meerbeek B. (2017). Biomechanical behavior of endodontically treated premolars using different preparation designs and CAD/CAM materials. *Journal of Dentistry*.

[29] Shinogaya T., Bakke M., Thomsen C. E., Vilmann A., Matsumoto M. (2000). Bite force and occlusal load in healthy young subjects—a methodological study. *European Journal of Prosthodontics and Restorative Dentistry*.

[30] Hagberg C. (1987). Assessments of bite force: a review. *Journal of Craniomandibular Disorders*.

[31] Bakke M., Michler L., Han K., Möller E. (1989). Clinical significance of isometric bite force versus electrical activity in temporal and masseter muscles. *European Journal of Oral Sciences*.

[32] Ferrario V. F., Sforza C., Serrao G., Dellavia C., Tartaglia G. M. (2004). Single tooth bite forces in healthy young adults. *Journal of Oral Rehabilitation*.

[33] Tortopidis D., Lyons M. F., Baxendale R. H., Gilmour W. H. (1998). The variability of bite force measurement between sessions, in different positions within the dental arch. *Journal of Oral Rehabilitation*.

[34] Behr M., Rosentritt M., Leibrock A., Schneider-Feyrer S., Handel G. (1999). In-vitro study of fracture strength and marginal adaption of fibre-reinforced adhesive fixed partial inlay dentures. *Journal of Dentistry*.

[35] Kern M., Strub J. R., Lü X.-Y. (1999). Wear of composite resin veneering materials in a dual-axis chewing simulator. *Journal of Oral Rehabilitation*.

[36] Monaco C., Krejci I., Bortolotto T., Perakis N., Ferrari M., Scotti R. (2006). Marginal adaptation of 1 fiber-reinforced composite and 2 all-ceramic inlay fixed partial denture systems. *International Journal of Prosthodontics*.

[37] Bankoğlu Güngör M., Turhan Bal B., Yilmaz H., Aydin C., Karakoca Nemli S. (2017). Fracture strength of CAD/CAM fabricated lithium disilicate and resin nano ceramic restorations used for endodontically treated teeth. *Dental Materials*.

