# Supplementary material for: Fracture Resistance of CAD/CAM Lithium Disilicate of Endodontically Treated Mandibular Damaged Molars Based on Different Preparation Designs
Source: Int J Biomater. 2019 May 12;2019:2475297. doi: 10.1155/2019/2475297 (PMC6535888; doi:10.1155/2019/2475297)
Supplement: Supplementary Materials — Fracture types: (1) detachment of the endocrown without fracture; (2) fracture without detachment of the endocrown and presence of a crack in the remaining tooth structure; (3) fracture with partial detachment of the endocrown and presence of a crack in the remaining tooth structure; (4) fracture with partial detachment of the endocrown and remaining tooth structure. [file 2475297.f1.docx]

May 1^st^, 2019

Dear Ms. Yassmin Mabrouk,

Please find enclosed the description of supplementary material.

I organized the figures and descriptions more concisely.


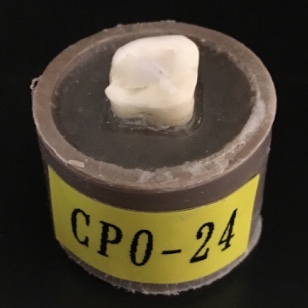

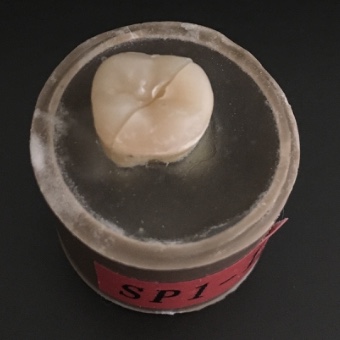

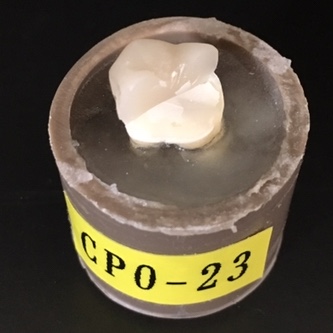

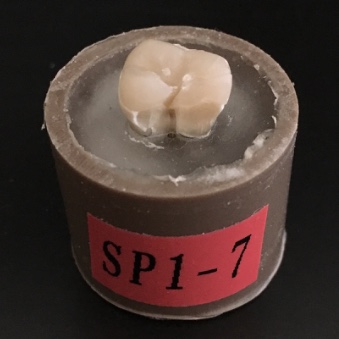


**1**

**2**

**3**

**4**

**Fracture types.**1, detachment of the endocrown without fracture; 2, fracture without detachment of the endocrown and presence of a crack in the remaining tooth structure; 3, fracture with partial detachment of the endocrown and presence of a crack in the remaining tooth structure; 4, fracture with partial detachment of the endocrown

and remaining tooth structure.

Thank you,

Sincerely,

Leandro Passos
